# *In Vitro* and *In Silico* Antioxidant Efficiency of Bio-Potent Secondary Metabolites From Different Taxa of Black Seed-Producing Plants and Their Derived Mycoendophytes

**DOI:** 10.3389/fbioe.2022.930161

**Published:** 2022-07-19

**Authors:** Abdallah M. A. Hassane, Saleh M. Hussien, Mohamed E. Abouelela, Taher M. Taha, Mohamed F. Awad, Hassan Mohamed, Mohammad M. Hassan, Mohammad H. A. Hassan, Nageh F. Abo-Dahab, Abdel-Rehim A. El-Shanawany

**Affiliations:** ^1^ Department of Botany and Microbiology, Faculty of Science, Al-Azhar University, Assiut, Egypt; ^2^ Food Science and Technology Department, Faculty of Agriculture, Al-Azhar University, Assiut, Egypt; ^3^ Department of Pharmacognosy, Faculty of Pharmacy, Al-Azhar University, Assiut, Egypt; ^4^ Department of Biology, College of Science and Arts, Al Bahah University, Al-Mandaq, Saudi Arabia; ^5^ Department of Biology, College of Science, Taif University, Taif, Saudi Arabia; ^6^ Colin Ratledge Center for Microbial Lipids, School of Agricultural Engineering and Food Science, Shandong University of Technology, Zibo, China; ^7^ Department of Genetics, Faculty of Agriculture, Menoufiya University, Sheben Al Kom, Egypt; ^8^ Department of Botany and Microbiology, Faculty of Science, Assiut University, Asyut, Egypt

**Keywords:** black seed plants, mycoendophytes, phenolics, flavonoids, antioxidant, HPLC, molecular docking

## Abstract

Oxidative stress is involved in the pathophysiology of multiple health complications, and it has become a major focus in targeted research fields. As known, black seeds are rich sources of bio-active compounds and widely used to promote human health due to their excellent medicinal and pharmaceutical properties. The present study investigated the antioxidant potency of various black seeds from plants and their derived mycoendophytes, and determined the total phenolic and flavonoid contents in different extracts, followed by characterization of major constituents by HPLC analysis. Finally, *in silico* docking determined their binding affinities to target myeloperoxidase enzymes. Ten dominant mycoendophytes were isolated from different black seed plants. Three isolates were then selected based on high antiradical potency and further identified by ITS ribosomal gene sequencing. Those isolated were *Aspergillus niger* TU 62, *Chaetomium madrasense* AUMC14830, and *Rhizopus oryzae* AUMC14823. *Nigella sativa* seeds and their corresponding endophyte *A. niger* had the highest content of phenolics in their *n-*butanol extracts (28.50 and 24.43 mg/g), flavonoids (15.02 and 11.45 mg/g), and antioxidant activities (90.48 and 81.48%), respectively, followed by *Dodonaea viscosa* and *Portulaca oleracea* along with their mycoendophytic *R. oryzae* and *C. madrasense*. Significant positive correlations were found between total phenolics, flavonoids, and the antioxidant activities of different tested extracts. The *n*-butanol extracts of both black seeds and their derived mycoendophytes showed reasonable IC_50_ values (0.81–1.44 mg/ml) compared to the control with significant correlations among their phytochemical contents. Overall, seventeen standard phenolics and flavonoids were used, and the compounds were detected in different degrees of existence and concentration in the examined extracts through HPLC analysis. Moreover, the investigation of the molecular simulation results of detected compounds against the myeloperoxidase enzyme revealed that, as a targeted antioxidant, rutin possessed a high affinity (−15.3184 kcal/mol) as an inhibitor. Taken together, the black seeds and their derived mycoendophytes are promising bio-prospects for the broad industrial sector of antioxidants with several valuable potential pharmaceutical and nutritional applications.

## 1 Introduction

Oxidants, represented by reactive oxygen or nitrogen species , as an output of metabolic processes, are causative of oxidative stress. These free radicals induce structural changes in cellular biomacromolecules, such as lipids, proteins, and nucleic acids, which eventually cause harm to cells (Apak et al., 2016). Living systems have varied modes of protection against free radicals, the efficacy of which is reduced by aging (Torre et al., 2019). Due to the accumulation of oxidants along with their affirmative reduction prospecting, many familiar disorders such as cancer, Alzheimer’s, renal failure, atherosclerosis, Parkinson’s, heart diseases, and diabetes have developed. The harmful impact of free radicals can be voided by antioxidants—molecules generated endogenously (enzymatic: catalase, superoxide dismutase, and gluthatione peroxidase; nonenzymatic: vitamin A) or provided exogenously (nonenzymatic: organic natural supplements) (Charmforoshan et al., 2019; Sajjadi et al., 2019; Munteanu and Apetrei, 2021).

Bio-efficient natural products offer a valuable source of novel antioxidants and antimicrobial agents, besides their minimal side effects and virtue therapeutic prospects (Farag and Swaby, 2018). Plants are known to produce a vast number of natural secondary metabolites with unique pharmacological potential; these by-products are phenols, alkaloids, and terpenoids (Tavassoli and Djomeh, 2011). Although a wide spectrum of microorganisms produce biopotent by-products, the research deriving these secondary metabolites has pivoted principally on medicinal plants (Amer, 2018).

Mycoendophytes are fungi that subsist within plant tissues without generating any adverse symptoms (Meenatchi et al., 2016). Diverse fungal endophytes biosynthesize biopotent by-products that imitate their host plant, along with having varied nutritional, industrial and medical applications (Sharma et al., 2016). Due to a high biodiversity and biochemical evolution, mycoendophytes have the capacity to utilize sundry substrates, with a broad output of natural products (Guanatilaka, 2006; Fernandes et al., 2009). These metabolites encompass alkaloids, phenols, steroids, terpenoids, and isocoumarins (Schulz et al., 2002). There have been few studies concerning the bioactivity of black seeds and their derived mycoendophytes. Extracts derived from black seeds using various kinds of organic solvents, as well as their endophytic fungi (Mohamed et al., 2021), possess a significant antioxidant (Ashraf et al., 2011; Metwaly et al., 2019), antibacterial (Youssef et al., 2015; Sabra and Ismail, 2019), antifungal (Rizzello et al., 2009; Khattak et al., 2020), and anticancer efficacy (Abdulmyanova et al., 2016; Leporini et al., 2018).

The polyphenolic compounds, particularly flavonoids, are reported to play a significant role in antioxidant defense mechanisms and provide protection against body damage by reactive oxygen species (ROS) (Shahidi and Ambigaipalan, 2015). Despite their great beneficial effects on human health, flavonoids cannot be biosynthesized in the human body, making diet the only means of uptake (Magiera et al., 2015). Thus, their existence in natural products, especially in foods and feeds, makes them an outstanding choice as a nutraceutical. Polyphenols are widespread in plants and fungi, in which they play different roles as structural and functional metabolites (Cimmino et al., 2013). Neoflavones, naturally occurring flavonoids (Jung et al., 2018), manifest antibacterial activities (Veselinovic et al., 2014). The catechin polyphenols can act on *S. aureus*, through destabilization and permeabilization of the cell membrane as well as inhibition of enzymes (Ferrazzano et al., 2011). Protocatechuic acid exhibits antifungal, anti-inflammatory, antiviral, anti-hepatotoxic, apoptotic, and neuroprotective activities (Khadem and Marles, 2010).

Computer-aided drug design and *in silico* assay technology minimize squandered time and economic load in the drug discovery process (Dincel et al., 2020). Molecular docking has been extensively employed to resolve interactions between bio-efficient molecules and proteins and to investigate their bioactivity (Kongpichitchoke et al., 2016). *N. sativa* (black seeds) is a widely used medicinal plant throughout the world. According to one of the Prophet Muhammad’s (PBUH) statements, “*The black seed can heal every disease, except death*” [Sahih Bukhari vol. 7 book 71 # 592] (Ishtiaq et al., 2013; Ali et al., 2018). Different black seeds obtained from different plant species, along with seeds of the common *N. sativa* plant, were investigated to: 1) identify mycoendophytes associated with black seeds that belong to different plant species; 2) evaluate the antioxidant potential of promising isolated strains along with their host plants; 3) determine the phenolic and flavonoid components responsible for antioxidant activity using HPLC analysis, and 4) analyze these components to determine their binding affinities to target myeloperoxidase, an enzyme that catalyzes the formation of reactive intermediates.

## 2 Materials and Methods

### 2.1 Collection of Plant Seeds

Ten different healthy black seed plants, including *Allium ampeloprasum*, *Allium cepa, Amaranthus retroflexus*, *Dodonaea viscose*, *N. sativa*, *Ocimum basilicum*, *Papaver somniferum*, *Piper nigrum*, and *Portulaca oleracea*, were collected from Assiut governorate (27°10′48.4824″N, 31°11′21.4188″E), Egypt, while *Pancratium maritimum* mature seeds were collected from the Alexandrian coast of the Mediterranean Sea in Egypt (30°56′51ʺN, 29°30′41ʺE) (Mohamed et al., 2018) for the isolation of endophytic fungi. Plant seeds were identified by plant taxonomy staff members based on reference books with species descriptions, keys, and illustrations. Black seed plant voucher specimens were deposited at the herbarium of the Botany and Microbiology Department, Faculty of Science, Al-Azhar University, Assiut, Egypt, with voucher numbers.

### 2.2 Isolation of Fungal Endophytes

The seeds were surface sterilized for the isolation of endophyic fungi, as described by Geisen et al. (2017) , with some modifications. The seeds were rinsed with sterile double distilled water for 5 min, and then sterilized in 5% sodium hypochlorite solution for 5 min, followed by soaking in ethanol 70% for 3 min. After that, the seeds were washed twice with sterile double distilled water and dried using filter papers under aseptic conditions. Individual seeds were inoculated into potato dextrose agar (PDA) medium, containing potato infusion 200 g/L, dextrose 20 g/L, and agar 20 g/L into 1,000 ml distilled H_2_O with final pH (5:6 ± 0:2), in addition to chloramphenicol and Rose Bengal, as bactericide and fast-growing fungi restrictor, respectively. In addition, 100 μl of the resulting washing water suspensions were streaked on PDA. All plates were incubated at 28 ± 2°C and monitored periodically for fungal development for up to 2 weeks. The growing fungal mycelia were picked, purified, and kept on PDA slants at 4°C for further investigation.

### 2.3 Identification of Fungal Isolates

The fungal cultures were characterized on the basis of their morphological features. The identified fungi were confirmed by Prof. Ahmed M. Moharram, Professor of Mycology, Botany and Microbiology Department, Faculty of Science, Assiut University, Egypt, and Head Manager of Assiut University Mycological Center (AUMC), and the most active selective isolates were confirmed using molecular biological protocols (Hassan et al., 2019; Mohamed et al., 2020), and deposited in the AUMC culture collection with institutional numbers.

### 2.4 Molecular Identification of the Selected Fungi

#### 2.4.1 Isolation of Genomic DNA

Czapek-Dox broth culture medium was employed to cultivate the pure fungi for 5 days at 25–28°C. The extraction procedures of the total DNA from each isolate were performed using a specific kit “Norgen Plant/Fungi DNA Isolation Kit (Sigma, Thorold, Canada)” according to Hassan et al. (2019). DNA was kept after elution at −20°C for further studies.

#### 2.4.2 PCR Amplification and Nucleotide Sequence Analysis

Specific universal primers ITS-1 (5′-TCC GTA GGT GAA CCT GCG G-3′) and ITS-4 (5′-TCC TCC GCT TAT TGA TAT GC-3′) were used in the present investigation to amplify the ITS region of the selected strains as previously suggested by Mohamed et al. (2020). PCR reaction was achieved as reported by Hassan et al. (2019), and the mixture of PCR tubes contained 25 μl, viz., 12.5 μl of PCR master mix, 1 μl from both forward and reverse primers (approx. 20 pmol), genomic DNA 1 and 9.5 μl of ddH_2_O. The amplification of DNA was carried out in Thermocycler C1000 Touch™ (Bio-Rad, Germany). The PCR products obtained were resolved on agarose gel 1.5% using TBE buffer, and visualized through a gel documentation system. Then the gel extraction kit (Omega Bio-tek) was applied to purify the PCR product based on the manufacturer’s instructions. All PCR products were sequenced with the gene analyzer 3121 sequencing service (Macrogen Co., Seoul, South Korea). The sequences of selected strains were aligned with the BLAST search tool at (NCBI) to detect sequence similarity. The obtained sequences were checked and analyzed using BioEdit software program version No. 7.2.5, and the homology search was carried out by comparison to isolates from the sequencing databases using a BLAST search algorithm of the GenBank (http://www.ncbi.nlm.nih.gov/BLAST).

### 2.5 Preparation of Extracts

#### 2.5.1 Propagation and Extraction of Fungal Metabolites

Propagation of isolated fungi was carried out by solid state fermentation (Al Mousa et al., 2021) on autoclaved 100 g rice and 100 ml H_2_O in 1L Erlenmeyer flasks. After cooling, 2 ml spore suspensions (10^5^ cfu/ml) were inoculated into flasks, and incubated at 28°C for 1 month. After that, the fermented rice was defatted by petroleum ether, and then sequential extraction of fungal metabolites was employed using gradual polar solvents, namely *n*-butanol, acetone and methanol, respectively, for 24 h at room temperature (Al-Fatimi et al., 2007). Each extract was filtered and dried up with a rotavapor (BÜCHI R-114, Switzerland).

#### 2.5.2 Preparation of Seeds Extracts

Dried seeds were powdered using an electric mill, and 30 g was defatted and extracted as described above. Each extract was filtered and dried up with a rotavapor (BÜCHI R-114, Switzerland) to yield the crude extract that was collected in a vial for further investigation.

### 2.6 Estimation of Total Phenolic Content

Total phenolic content assay was assessed using modified procedures of Suleria et al. (2012). In brief, 0.5 ml of the specimen (10 mg/ml) was added to the same volume of Folin–Ciocalteu’s phenol reagent, followed by the addition to the reaction solution of 1 ml of 10% Na_2_CO_3_ after 3 min. Incubation of the mixture proceeded under shaking and dark conditions at 180 rpm for 60 min at 25°C. Measurement of the absorbance was at 750 nm. Phenolic content was declared as gallic acid equivalent (GAE) (mg/g) through the later equation due to the standardization curve: *y* = 0.0169*x* − 0.1172, *R* ^2^ = 0.9588. The standard curve of gallic acid was linear between 0.5 and 100 μg/ml.

### 2.7 Determination of Total Flavonoid Content

A previous method reported by Quettier-Deleu et al. (2000) was conducted to measured total flavonoid content; 0.5 ml of each extract (10 mg/ml) was carefully mixed with 1.0 ml of a 2% (v/v) AlCl_3_.6H_2_O ethanolic solution, and the absorbance was measured at 430 nm after 10 min. The total flavonoid content was explicated as quercetin equivalent (QE) (mg/g) by employing the later equation due to the standardization curve: *y* = 0.0208*x* − 0.2381, *R* ^2^ = 0.9678. The calibration curve of quercetin was linear between 0.5 and 100 μg/ml.

### 2.8 Antioxidant Activity Assay

#### 2.8.1 DPPH Scavenging Activity (%) Assay

The antioxidant properties of the test samples were measured for scavenging activity or hydrogen donating form based on the procedure reported before (Brand-Williams et al., 1995). While the DPPH radical was scavenged, the color changed from purple to yellow with a 517 nm absorbance decrease. Then, 1.8 ml of 0.1 mM DPPH (Sigma-Aldrich, Germany) (4 mg/100 ml of methanol) mixed solution was added to 0.2 ml of the tested samples in absolute methanol at various concentrations (1, 0.8, 0.6, 0.4, 0.2, 0.1, and 0.05 mg/ml) beside the blank. The mixture was left aside at room temperature (shaken vigorously in-between) for 30 min and absorbance was determined by spectrophotometer (Jenway 7315) at 517 nm. Butylated hydroxytoluene (BHT) was employed as a positive control and all measurements were performed in triplicate. The following formula was used to determine the capacity to scavenge the DPPH radical

$$\text{\% DPPH radical scavenging }=\frac{\text{A}-B}{\text{A}}\;\times 100$$ (1)

where A is the negative control absorbance (methanol and DPPH) and B is the sample absorbance (DPPH, methanol and sample)*.* The IC_50_ was obtained by interpolation from linear regression analysis, where the obtained regression equation was *y* = a*x* + b, IC_50_ was calculated as IC_50_ = (0.5 − b)/a. The IC_50_ value denotes the level of the antioxidant capacity of the tested extracts. The IC_50_ value is inversely proportional to the free radical scavenging property of the sample.

#### 2.8.2 Hydrogen Peroxide Scavenging Activity Assay

The IC_50_ values of the most potent extracts were investigated according to the method described by Ruch et al. (1989), where the extracts at various concentrations (1, 0.8, 0.6, 0.4, 0.2, 0.1, and 0.05 mg/ml) beside the blank were added to a hydrogen peroxide solution (0.6 ml, 40 mM) and the absorbance at 230 nm was determined 10 min later against a blank solution containing the phosphate buffer. The percentage of hydrogen peroxide scavenging of the extracts were calculated: % Scavenged [H_2_O_2_] = [(A0 − A1)/A0] × 100, where A0 was the control absorbance and A1 the absorbance of the sample.

#### 2.8.3 Nitric Oxide Scavenging Activity Assay

The scavenging impact of the most potent extracts on nitric oxide (NO) was determined according to Sreejayan and Rao (1997). Sodium nitroprusside (10 mM) was mixed with extracts (1, 0.8, 0.6, 0.4, 0.2, 0.1, and 0.05 mg/ml) and incubated for 150 min. After that, the test was mixed with 0.5 ml of Griess reagent and then measured at 546 nm. In the control, sample extract was substituted by PBS. The capability of scavenging NO was calculated using the following equation: Scavenging effect (%) = [1 − (A sample/A control)] × 100.

### 2.9 High Performance Liquid Chromatography Analysis of Flavonoid and Phenolic Contents

The principal flavonoids and phenolic compounds were detected and identified using an Agilent 1260 series HPLC system with an Eclipse C18 column (4.6 mm × 250 mm i.d., 5 μm) and water (A) and 0.05% trifluoroacetic acid in acetonitrile (B) as the mobile phase at a flow rate of 1 ml/min. The mobile phase elution linear gradient was used as follows: 0 min (82% A:18% B); 0–5 min (80% A:20% B); 5–8 min (60% A:40% B); 8–12 min (60% A:40% B); and 12–20 min (82% A:18% B). The multi-wavelength detector used was monitored at a wavelength of 280 nm. The injection volume for each sample was 5 μl and the column temperature was maintained at 40°C. Each sample’s phenolic and flavonoid composition was determined by comparing their retention times and spectral reference data with the external standard controls. All standards, namely gallic acid, chlorogenic acid, catechin, methyl gallate, caffeic acid, syringic acid, pyrocatechol, rutin, ellagic acid, coumaric acid, vanillin, ferulic acid, naringenin, querectin, cinnamic acid, kaempferol, and hesperetin were purchased from Sigma-Aldrich (Khalil et al., 2020; Abdel-Aziz et al., 2021).

### 2.10 Molecular Docking Simulations With Antioxidant Target Protein

Molecular Operating Environment software (MOE 2019.01) was utilized for optimization of both examined receptors and compounds for docking study. The inhibition of *in vivo* production of reactive oxygen species (ROS) by the seventeen detected compounds was assessed by evaluating their ligand-protein binding patterns and interactions with myeloperoxidase enzymatic protein (1DNU), retrieved from the Protein Data Bank (https://www.rcsb.org/pdb) (Vadabingi et al., 2020).

The target protein was prepared for docking by Quickprep function and removing unnecessary water molecules and all co-crystallized ligands and metals. The active site for the interactions was selected by site finder built-in function. Further, the detected compounds were subjected to 3D generation, 3D potentiation energy minimization using Merck Molecular Forcefield (MMFF94x), and stochastic conformational search. The conformers with minimum energy were selected and a virtual ligand database was generated. The rigid receptor-flexible ligand was adopted as the docking procedure using Triangle Matcher placement and rigid receptor refinement with London dG as the scoring and rescoring algorithm retaining 10 poses. The docking score and root mean square deviation (RSMD) were recorded. 2D and 3D interaction figures were generated by the MOE visualization tool (Gabr et al., 2019).

### 2.11 Data Analysis

All experiments and measurements were performed thrice. Using the SPSS, software program (version No. 16), and one-way ANOVA, the values were expressed as the mean ± SE at the 0.05 significance level.

## 3 Results

### 3.1 Fungal Isolates and Their Sequence Data

Based on the phenotypic and microscopic cultural characteristics, ten dominant endophytic fungi belonged to four genera and seven species were isolated from black seeds of ten different plant taxa including: *Aspergillu*s amstelodami AUMC14827 from *O. basilicum*; *A. niger* AUMC14825 and 14829 from *A. ampeloprasum* and *N. sativa*, respectively; *A. versicolor* AUMC14828 from *P. nigrum*; *C. madrasense* AUMC14830 from *P. oleracea*; *Penicillium chrysogenum* AUMC14822, 14831 and 14826 from *A. cepa*, *P. maritimum*, and *P. somniferum*, respectively; *P. citrinum* AUMC14824 from *A. retroflexus*; and *R. oryzae* AUMC14823 from *D. viscose* (Table 1).

**TABLE 1:** Endophytic fungi isolated from different black seed plants.

AUMC^a^ no.	Endophytic fungi	Phylum; class; order	Host plant	Common name	Herbarium voucher no.	Plant family
14827	*A. amstelodami* (Mangin) Thom & Church	Ascomycota; Eurotiomycetes; Eurotiales	*O. basilicum* L.	Basil	ABH308-6	Lamiaceae
14825	*A. niger* van Tieghem		*A. ampeloprasum* L.	Leek	ABH20-29	Amaryllidaceae
14829	*A. niger* van Tieghem		*N. sativa* L.	Black seed	ABH490-14	Ranunculaceae
14828	*A. versicolor* (Vuillemin) Tiraboschi.		*P. nigrum* L.	Black Pepper	ABH449-574	Piperaceae
14830	*C. madrasense* Natarajan	Ascomycota; Sordariomycetes; Sordariales	*P. oleracea* L.	Purslane	ABH470-36	Portulacaceae
14822	*P. chrysogenum* Thom	Ascomycota; Eurotiomycetes; Eurotiales	*A. cepa* L.	Onion	ABH20-143	Amaryllidaceae
14831	*P. chrysogenum* Thom		*P. maritimum* L.	Sand lily	ABH20-10	Amaryllidaceae
14826	*P. chrysogenum* Thom		*P. somniferum* L.	Opium poppy	ABH421-46	Papaveraceae
14824	*P. citrinum* Thom		*A. retroflexus* L.	Redroot pigweed	ABH19-76	Amaranthaceae
14823	*R. oryzae* Went & Prinsen-Geerligs	Zygomycota; Zygomycetes; Mucorales	*D. viscosa* L.	Hopbush	ABH518-66	Sapindaceae

a **AUMC**, Assiut University Mycological Center.

**FIGURE 1:**
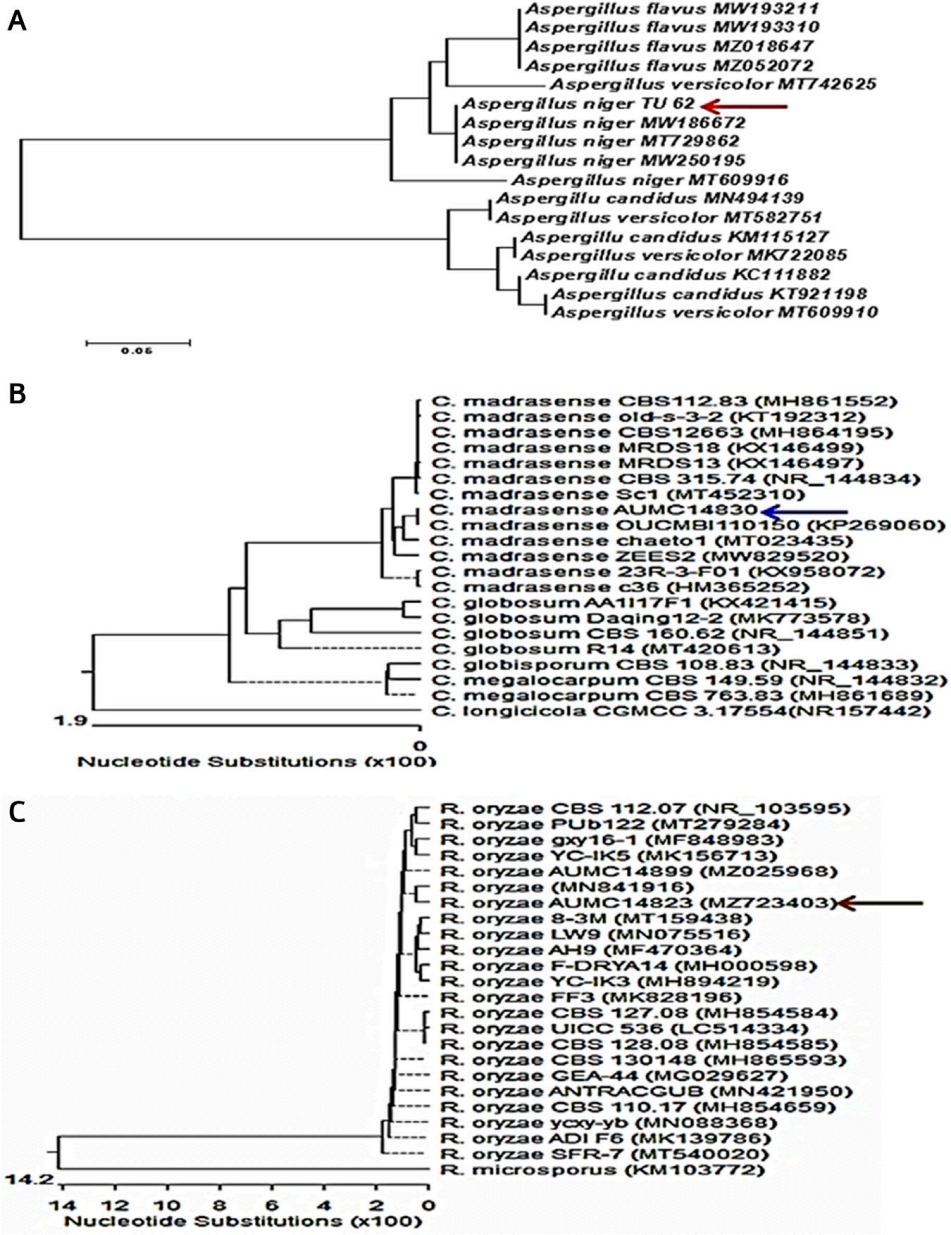
Neighbor-joining phylogenetic tree showing the genetic diversity of fungal isolates based on ITS sequences of tested fungi aligned with closely-related strains accessed from GenBank: **(A)** *A. niger* TU62; **(B)** *C. madrasense* AUMC14830; **(C)** *R. oryzae* AUMC14823.

Identification of the most potent isolates was done by performing the ITS region sequencing, and the obtained sequences were further subjected to a BLAST search at NCBI database. The isolates were confirmed as *A. niger* TU 62 (GenBank accession no. OL411611)*,* C. *madrasense* AUMC14830 (GenBank accession no. OL588256), and *R. oryzae* AUMC14823 (GenBank accession no. MZ723403) (Figure 1). Nucleotide comparisons of the ITS regions among *Aspergillus* strains and other similar strains retrieved from NCBI revealed 100% identity between *A. niger* TU 62 and each of *A. niger* MW186672 and *A. niger* MT729862 from GenBank. Moreover, *C. madrasense* AUMC14830 showed 99.12%–100% identity with several strains of *C. madrasense*, including the type strain CBS315.74 with GenBank accession no. NR_144834. At the same time, the sequenced *R. oryzae* AUMC14823 showed 100% identity and 100% coverage with several strains of *R. oryzae*, including the type material *R. oryzae* CBS 112.07 with GenBank accession no. 103595.

### 3.2 Determination of Total Phenolics, Flavonoids, and Antioxidant Activity of Extracts

**FIGURE 2:**
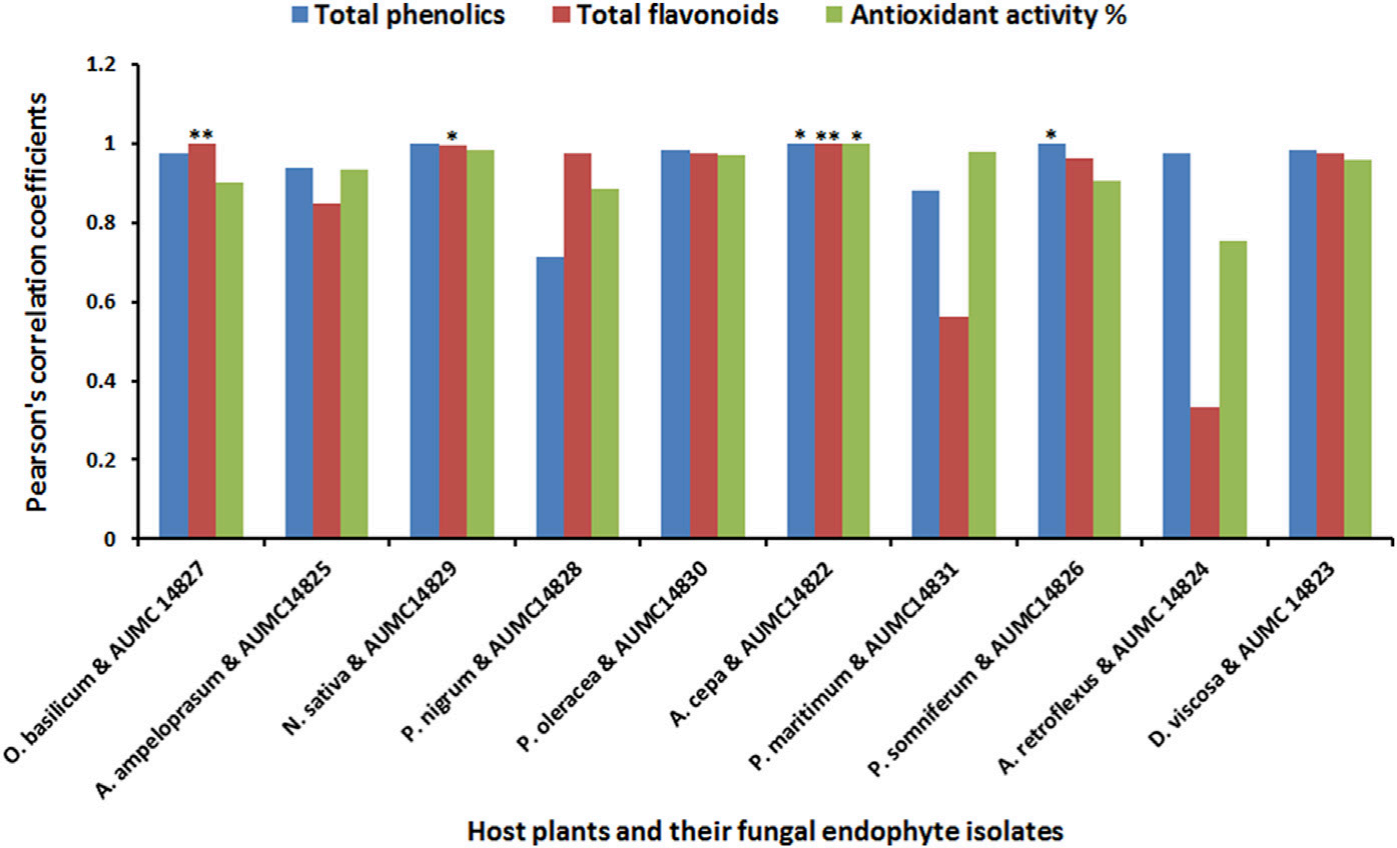
Pearson’s correlation coefficients of total phenolics, flavonoids, and the antioxidant activities of different extracts from black seed plants and their mycoendophytic isolates.

The present study aimed to determine the antioxidant activity of *n*-butanol, acetone, and methanol extracts obtained from ten black seed plants and their associated endophytic fungi. Total phenolics and flavonoids, which are closely correlated to antioxidant potency, were investigated in all extracts. The results indicated that butanolic extracts of both the seeds and their endophytic fungi had higher content of total phenolics, flavonoids, and antioxidant activity than acetonic and methanolic extracts except for *O. basilicum*, *P. maritimum*, and *A. retroflexus* along with their fungal endophytes. The highest content of phenolics in butanolic extracts was found in *N. sativa* and *D. viscosa* (28.50 and 26.51 mg/g, respectively), while their corresponding endophytes *A. niger* and *R. oryzae* contained 24.43and 24.40 mg/g, respectively. Elevated content of total flavonoids were recorded in the *n*-butanol extracts of *N. sativa* and *P. oleracea* (15.02 and 10.46 mg/g) and their associated endophytic *A. niger* and *C. madrasense* (11.45 and 9.23 mg/g), respectively. Moreover, *N. sativa* and its endophytic *A. niger* exhibited the best antioxidant activities (90.48 and 81.48%), followed by *D. viscosa* and its accompanying *R. oryzae* (89.21 and 81.71%), whereas *P. oleracea* and its partner *C. madrasense* got 81.42 and 78.55%, respectively, followed by *P. somniferum* and derived *P. chrysogenum* (Table 2). Regarding Pearson’s correlation of total phenolics, flavonoids, and the antioxidant activities of different organic solvent extracts from plant seeds and their derived fungi, positive correlations were revealed along with significant correlations between some parameters (Figure 2).

**TABLE 2:** Pearson’s correlation coefficients of total phenolics, flavonoids, and antioxidant activities by DPPH assay of *n*-butanol, acetone and methanol extracts from the investigated host, black seed plants, and their endophytic fungal isolates.

Host plants and their fungal endophyte isolates	Extracts									Pearson’s correlation coefficients		
	Butanol			Acetone			Methanol			Total phenolics	Total flavonoids	Antioxidant activity %
	Total phenolics (mg/gm)	Total flavonoids (mg/gm)	Antioxidant activity %	Total phenolics (mg/gm)	Total flavonoids (mg/gm)	Antioxidant activity %	Total phenolics (mg/gm)	Total flavonoids (mg/gm)	Antioxidant activity %			
*O. basilicum*	15.12 ± 0.04	6.74 ± 0.02	62.30 ± 0.07	11.63 ± 0.21	5.48 ± 0.01	63.21 ± 0.03	15.53 ± 0.09	4.01 ± 0.08	65.85 ± 0.05	0.977	1.00**	0.903
AUMC14827	10.54 ± 0.03	4.73 ± 0.05	58.28 ± 0.04	8.64 ± 0.41	4.48 ± 0.01	39.01 ± 0.02	11.41 ± 0.07	4.20 ± 0.03	63.21 ± 0.03			
*A. ampeloprasum*	18.75 ± 0.00	5.74 ± 0.04	70.17 ± 0.12	13.83 ± 0.06	4.36 ± 0.01	58.63 ± 0.02	7.43 ± 0.08	4.11 ± 0.02	20.03 ± 0.23	0.937	0.847	0.936
AUMC14825	18.04 ± 0.07	5.70 ± 0.03	65.80 ± 0.03	11.12 ± 0.04	4.45 ± 0. 13	35.63 ± 0.06	8.81 ± 0.06	4.96 ± 0.20	11.03 ± 0.15			
*N. sativa*	28.50 ± 0.01	15.02 ± 0.14	90.48 ± 0.12	4.52 ± 0.06	3.47 ± 0.11	23.81 ± 0.02	7.27 ± 0.01	3.67 ± 0.21	32.95 ± 0.03	1.00	0.998*	0.984
AUMC14829	24.43 ± 0.10	11.45 ± 0.01	81.48 ± 0.04	2.23 ± 0.07	2.10 ± 0.04	21.70 ± 0.11	5.16 ± 0.13	2.85 ± 0.13	40.13 ± 0.08			
*P. nigrum*	19.34 ± 0.02	9.70 ± 0.05	78.32 ± 0.08	4.30 ± 0.07	2.55 ± 0.11	27.31 ± 0.03	3.17 ± 0.06	3.01 ± 0.22	16.25 ± 0.22	0.715	0.974	0.884
AUMC14828	12.15 ± 0.11	8.03 ± 0.06	71.21 ± 0.03	9.96 ± 0.15	3.00 ± 0.03	53.44 ± 0.08	2.20 ± 0.18	1.90 ± 0.18	25.23 ± 0.09			
*P.oleracea*	12.04 ± 0.16	10.46 ± 0.05	81.42 ± 0.08	9.92 ± 0.02	8.36 ± 0.11	52.93 ± 0.03	10.60 ± 0.80	8.01 ± 0.18	54.96 ± 0.05	0.984	0.975	0.970
AUMC14830	9.51 ± 0.10	9.23 ± 0.04	78.55 ± 0.01	8.92 ± 0.25	6.66 ± 0.01	46.83 ± 0.14	9.01 ± 0.23	6.92 ± 0.10	52.93 ± 0.03			
*A. cepa*	18.10 ± 0.05	7.73 ± 0.01	62.12 ± 0.11	7.70 ± 0.13	3.23 ± 0.12	21.36 ± 0.02	8.41 ± 0.11	4.65 ± 0.18	31.48 ± 0.21	0.999*	1.00**	0.999*
AUMC14822	17.59 ± 0.03	5.99 ± 0.08	54.06 ± 0.03	6.71 ± 0.06	1.12 ± 0.23	16.60 ± 0.08	7.86 ± 0.21	2.54 ± 0.04	27.44 ± 0.12			
*P. maritimum*	8.31 ± 0.11	3.79 ± 0.04	21.03 ± 0.10	12.31 ± 0.07	3.53 ± 0.08	63.65 ± 0.02	7.28 ± 0.01	2.51 ± 0.02	68.34 ± 0.06	0.883	0.563	0.978
AUMC14831	8.82 ± 0.12	1.18 ± 0.23	16.64 ± 0.12	10.10 ± 0.06	4.08 ± 0.11	42.60 ± 0.12	9.20 ± 0.02	3.61 ± 0.05	39.24 ± 0.15			
*P. somniferum*	17.90 ± 0.04	6.34 ± 0.13	81.40 ± 0.04	14.74 ± 0.03	4.94 ± 0.02	61.45 ± 0.02	10.56 ± 0.04	4.38 ± 0.09	63.48 ± 0.17	0.999*	0.962	0.906
AUMC14826	16.17 ± 0.07	5.08 ± 0.04	78.21 ± 0.02	13.20 ± 0.03	3.22 ± 0.03	41.42 ± 0.08	8.53 ± 0.02	3.21 ± 0.05	22.16 ± 0.06			
*A. retroflexus*	7.24 ± 0.04	3.21 ± 0.02	22.12 ± 0.04	15.54 ± 0.01	2.22 ± 0.04	70.91 ± 0.01	8.45 ± 0.03	1.38 ± 0.09	30.99 ± 0.11	0.977	0.334	0.756
AUMC 14824	5.85 ± 0.06	1.84 ± 0.03	19.30 ± 0.21	12.50 ± 0.05	5.31 ± 0.07	25.82 ± 0.15	7.34 ± 0.04	2.87 ± 0.05	12.34 ± 0.10			
*D. viscosa*	26.51 ± 0.02	8.14 ± 0.11	89.21 ± 0.04	13.56 ± 0.11	4.33 ± 0.02	40.34 ± 0.03	9.30 ± 0.13	3.28 ± 0.12	46.72 ± 0.04	0.983	0.974	0.961
AUMC 14823	24.40 ± 0.12	6.26 ± 0.02	81.71 ± 0.04	8.14 ± 0.23	2.43 ± 0.34	32.23 ± 0.07	7.12 ± 0.08	2.52 ± 0.26	22.13 ± 0.15			

The data were given as averages of three replicates (Mean ± SE).

*Correlation is significant at the 0.05 level (2-tailed).

**Correlation is significant at the 0.01 level (2-tailed).

The IC_50_ values determined for *n*-butanol extracts showed the highest antioxidant potency of both the seeds and their companion endophytic fungi using DPPH, H_2_O_2_ and nitric oxide assays. The positive control (BHT) presented IC_50_ values of 0.34, 0.41, and 0.33 mg/ml for DPPH, H_2_O_2_, and nitric oxide radicals, respectively. The data presented in Table 3 report that assayed *n*-butanol extracts demonstrated significant IC_50_ values for *N. sativa* along with its endophyte *A. niger*, introducing IC_50_ values toward DPPH free radicals of 0.81 and 1.16 mg/ml, followed by IC_50_ values of *D. viscosa* and its accompanying *R. oryzae* (1.11 and 1.28 mg/ml), whereas *P. oleracea* and its partner *C. madrasense* got 1.16 and 1.31 mg/ml, respectively. IC_50_ values induced by *N. sativa* and *A. niger* against H2O2 were 1.03 and 1.31 mg/ml, followed by *D. viscosa* and *R. oryzae* (1.31 and 1.44 mg/ml), while *P. oleracea* and *C. madrasense* attained IC_50_ values of 1.44 and 1.48 mg/ml, respectively. Moreover, the *N. sativa* and *A. niger* butanol extract versus nitric oxide radicals achieved IC_50_ values of 0.81 and 1.33 mg/ml, tailed by *P. oleracea* and *C. madrasense* (1.30 and 1.36 mg/ml), and *D. viscosa* along with *R. oryzae* (1.33 and 1.44 mg/ml), respectively. When compared with BHT, the *N. sativa* extract presented good IC_50_ values for antiradical activity within all assays, followed by the rest of the extracts which showed reasonable values. Our results showed significant positive correlations between different antioxidant activity assays of *n*-butanol extracts from seeds and associated mycoendophytic strains.

**TABLE 3:** IC_50_ values (mg/ml) and Pearson’s correlation coefficients of three antioxidant activity assays of butanol extracts from three black seed plants and associated endophytic fungal strains.

Extracts	IC_50_ values (mg/ml)			Pearson’s correlation coefficients		
	DPPH	H_2_O_2_	Nitric oxide	DPPH	H_2_O_2_	Nitric oxide
*N. sativa*	0.81 ± 0.07^c^	1.03 ± 0.05^c^	0.81 ± 0.02^c^	1	0.963**	0.945**
AUMC14829	1.16 ± 0.06^b^	1.31 ± 0.08^b^	1.33 ± 0.01^b^			
*P. oleracea*	1.16 ± 0.09^b^	1.44 ± 0.09^a^	1.30 ± 0.03^b^	0.963**	1	0.913*
AUMC14830	1.31 ± 0.02^a^	1.48 ± 0.04^a^	1.36 ± 0.02^b^			
*D. viscosa*	1.11 ± 0.09^b^	1.31 ± 0.01^b^	1.33 ± 0.10^b^	0.945**	0.913*	1
AUMC14823	1.28 ± 0.07^a^	1.44 ± 0.06^a^	1.44 ± 0.06^a^			
BHT	0.34 ± 0.05^d^	0.41 ± 0.04^d^	0.33 ± 0.07^d^			

The data were given as averages of three replicates (Mean ± SE). Values followed by the different letters are significantly different at *p* ˂ 0.05.

*Correlation is significant at the 0.05 level (2-tailed).

**Correlation is significant at the 0.01 level (2-tailed).

### 3.3 HPLC Analysis of Flavonoid and Phenolic Contents

**FIGURE 3:**
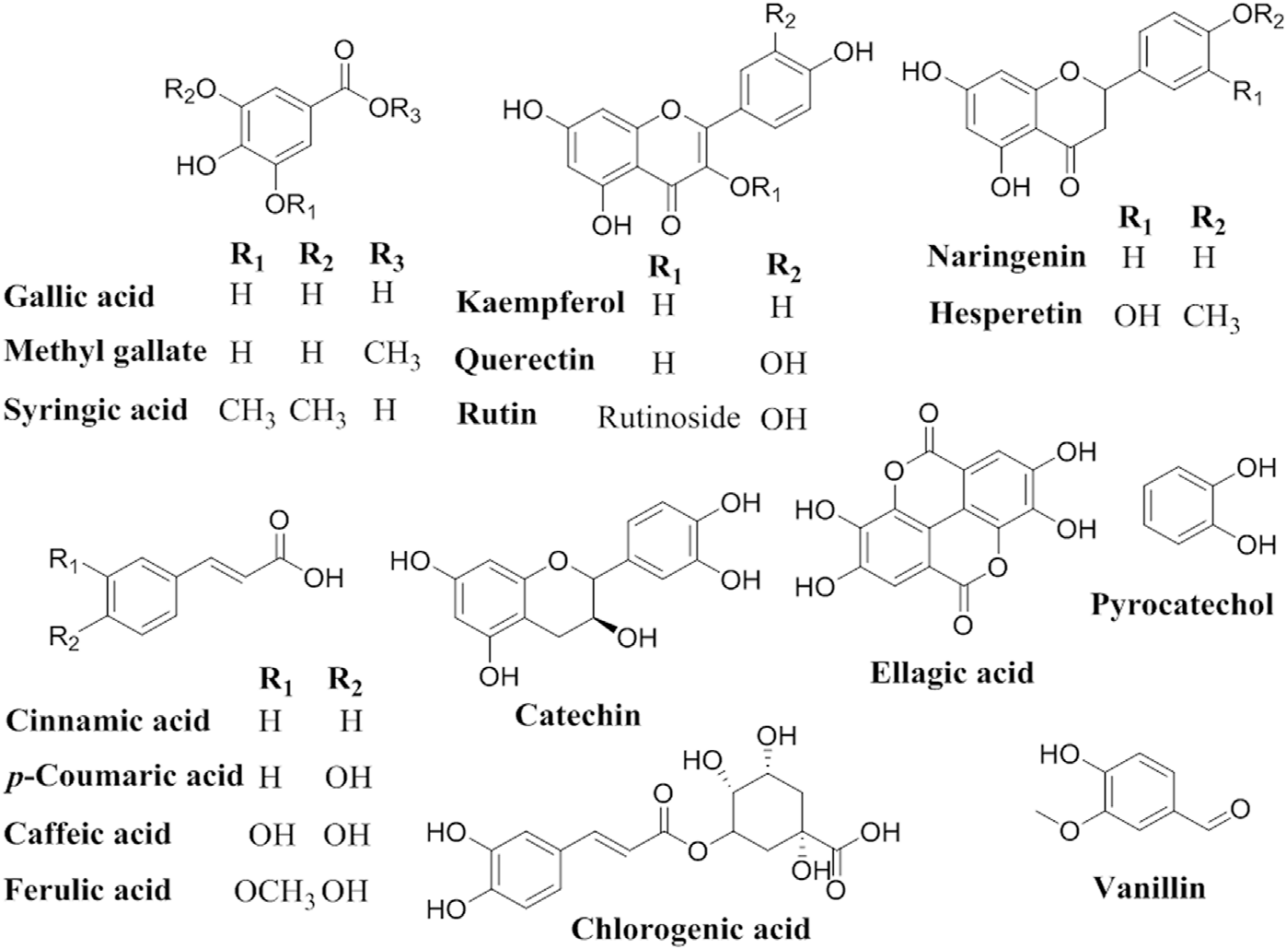
Chemical structures of detected compounds in different extracts.

**FIGURE 4:**
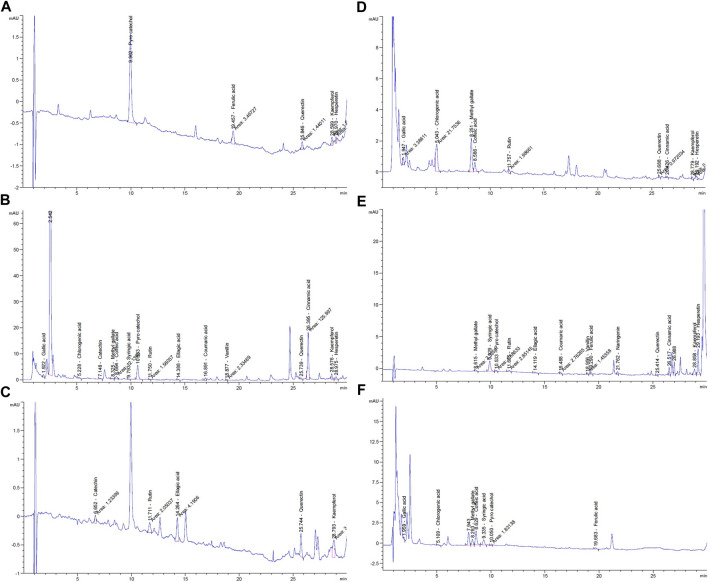
HPLC chromatograms of phenolic components in *n*-butanol extracts from: **(A)** *N. sativa* seeds; **(B)** endophytic fungus *A. niger*; **(C)** *P. oleracea* seeds; **(D)** endophytic fungus *C. madrasense;* **(E)** *D. viscosa* seeds; **(F)** endophytic fungus *R. oryzae*.

HPLC analysis for identification of the chemical constituents of the *n-*butanol extract of *N. sativa*, *P. oleracea*, and *D. viscosa*, and their derived endophytic fungi (*A. niger*, *C. madrasense*, and *R. oryzae*, respectively) was performed to correlate their potential antioxidant activities with these chemical ingredients. Seventeen standard phenolics and flavonoids, namely gallic acid, chlorogenic acid, catechin, methyl gallate, caffeic acid, syringic acid, pyrocatechol, rutin, ellagic acid, coumaric acid, vanillin, ferulic acid, naringenin, querectin, cinnamic acid, kaempferol, and hesperetin, were used in our study and the detected compounds are illustrated in Figure 3. The HPLC analysis results (Table 4; Figures 4A–F) depict the presence of varying phenolics and flavonoid amounts based on the above-mentioned standards in the tested extracts.

**TABLE 4:** Retention time (*R*_*t*_), and area percentage of detected phenolic and flavonoid compounds derived from selected black seeds and their associated fungi.

Peak	RT (min)	Standard compounds	Area %					
			*N. sativa*	*A. niger*	*P. oleracea*	*C. madrasense*	*D. viscosa*	*R. oryzae*
1	1.9	Gallic acid	ND^a^	0.49	ND	5.97	ND	2.71
2	5.1	Chlorogenic acid	ND	0.32	ND	38.34	ND	3.37
3	7.0	Catechin	ND	0.25	8.38	ND	ND	ND
4	8.6	Methyl gallate	ND	0.26	ND	34.41	2.65	11.57
5	8.7	Caffeic acid	ND	0.88	ND	10.57	ND	33.22
6	9.9	Syringic acid	ND	0.09	ND	ND	21.43	13.93
7	10.5	Pyrocatechol	75.79	3.78	ND	ND	1.80	3.63
8	11.7	Rutin	ND	0.11	13.93	3.50	3.24	ND
9	14.1	Ellagic acid	ND	0.11	28.47	ND	1.97	ND
10	16.5	*p*-Coumaric acid	ND	0.58	ND	ND	3.13	ND
11	19.0	Vanillin	ND	0.17	ND	ND	1.65	ND
12	19.3	Ferulic acid	10.88	ND	ND	ND	2.40	3.08
13	21.8	Naringenin	ND	ND	ND	ND	2.71	ND
14	25.4	Quercetin	4.52	0.23	23.55	1.71	1.00	ND
15	26.5	Cinnamic acid	ND	9.15	ND	1.09	7.75	ND
16	28.9	Kaempferol	5.21	0.89	25.67	1.66	9.35	ND
17	29.2	Hesperetin	3.60	0.56	ND	2.74	27.69	ND

a Not detected.

In this experiment, the *N. sativa* extract revealed the presence of pyrocatechol as a major compound (75.79%), reported for the first time in *N. sativa*, while its endophytic fungus *A. niger* showed its presence in a small amount (3.78%) and cinnamic acid was the predominant compound in the *A. niger* extract*.* As presented in Table 4, a total of five polyphenols were identified from seeds of *P. oleracea* using HPLC, namely; catechin, rutin, ellagic acid, quercetin, and kaempferol. The chemical analysis revealed the presence of ellagic acid 28.47%, kaempferol (25.67%), and quercetin (23.55%) as the main components, whereas chlorogenic acid (38.34%) and methyl gallate (34.41%) were found in *C. madrasense* endophyte and not detected in the host plant, indicating that it may be produced through biotransformation of host metabolites. On the other hand, methyl gallate, syringic acid, pyrocatechol, and ferulic acid were found in different proportions in both extracts of *D. viscosa* and its derived endophytic fungus *R. oryzae*. Hesperetin and syringic acid were the major detected compounds (27.69 and 21.43%, respectively) in the *D. viscosa* butanolic extract, while the endophytic fungi *R. oryzae* extract showed the presence of caffeic acid (33.22%) as a major compound not detected in the host plant.

### 3.4 *In Silico* Molecular Docking of Identified Compounds

According to the HPLC analysis, the different extracts of host plants and their endophytic fungi contained 17 polyphenolic compounds (Table 4). The docking studies for those compounds were analyzed to determine their binding affinities to target protein as antioxidants. Myeloperoxidase is an enzyme that catalyzes the formation of reactive oxygen intermediates and the generation of reactive nitrating and halogenating agents. It acts as a local tissue damage mediator in many inflammatory diseases. The investigation of molecular simulation results of detected phytomolecules against myeloperoxidase enzymatic protein (1DNU chain A & C) revealed that these compounds possess various affinities with the enzyme for acting as inhibitors, ranging from −15.3184 kcal/mol (rutin) to −6.1162 kcal/mol (vanillin) (Table 5). Therefore, they could have antioxidant effect by preventing reactive species release. The top scoring compounds were rutin, chlorogenic acid, quercetin, and kaempferol with pose scores of −15.3184 (RMSD = 0.99 Å), −12.1866 (RMSD = 0.64 Å), −10.6613 (RMSD = 0.76 Å), and −10.6037 (RMSD = 0.70 Å) kcal/mol, respectively.

**TABLE 5:** Pose score results of detected compound interactions with myeloperoxidase antioxidant target proteins.

No.	Name	Pose score (kcal/mol)	RMSD refine Å
1	Gallic acid	−8.3189	1.12
2	Chlorogenic acid	−12.1866	0.64
3	Catechin	−10.0794	1.22
4	Methyl gallate	−10.1663	1.18
5	Caffeic acid	−8.6657	0.57
6	Syringic acid	−9.6642	1.00
7	Pyrocatechol	−9.9019	1.13
8	Rutin	−15.3184	0.99
9	Ellagic acid	−9.8865	0.85
10	p-Coumaric acid	−7.0201	0.86
11	Vanillin	−6.1162	0.65
12	Ferulic acid	−7.8350	0.66
13	Naringenin	−9.0508	1.92
14	Quercetin	−10.6613	0.76
15	Cinnamic acid	−8.9470	1.29
16	Kaempferol	−10.6037	0.70
17	Hesperetin	−9.0758	1.03

**FIGURE 5:**
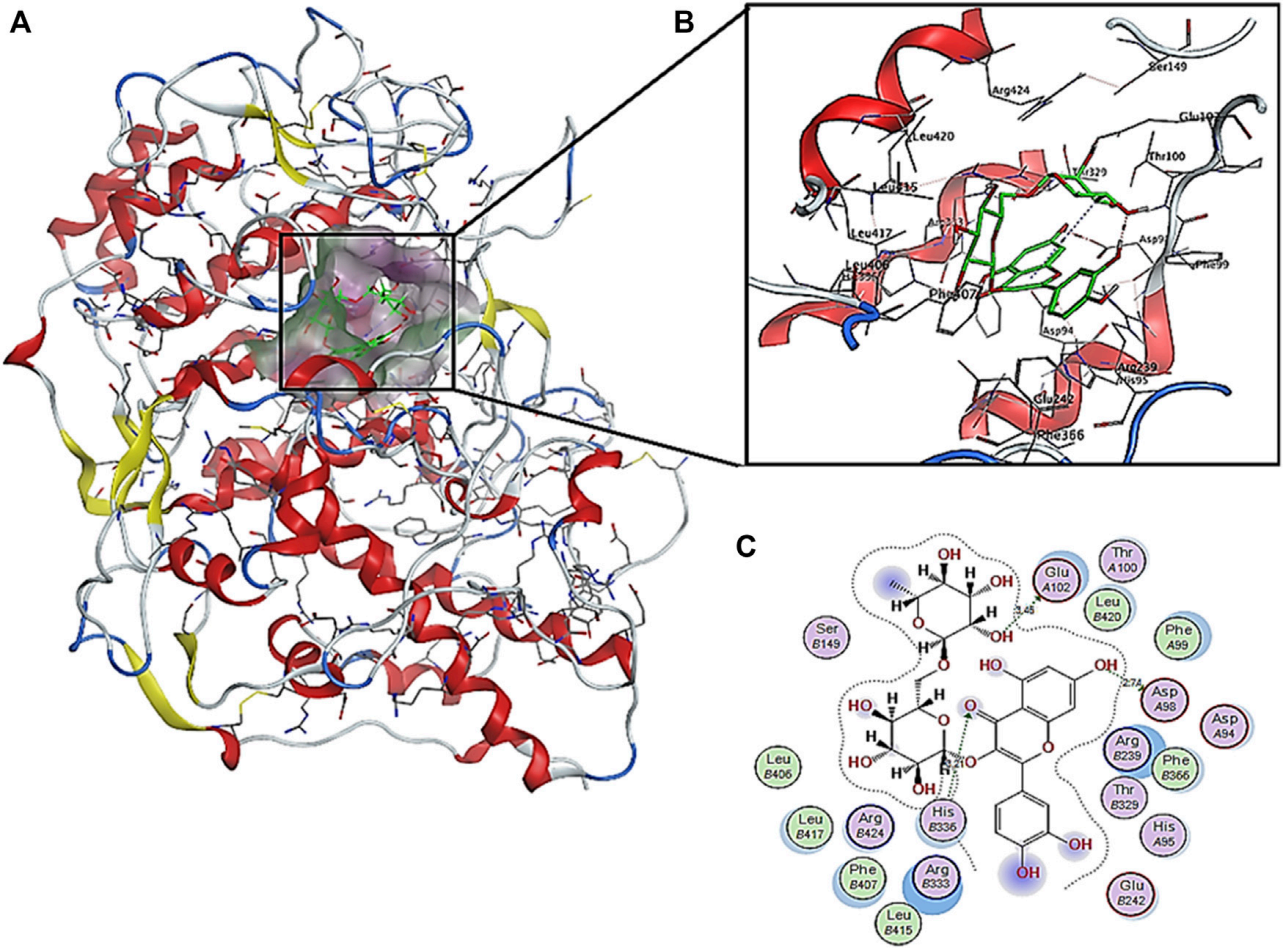
Predicted binding pose interactions with the residues of myeloperoxidase antioxidant target proteins: **(A)** whole protein binding site; **(B)** 3D interaction model; **(C)** 2D interaction model.

The interactions of the highest affinity compound (rutin) showed the presence of hydrogen bond interaction as H-donar with Glu A102 and Asp A98 and H-acceptor with His C 336 amino acid residues (Figure 5). In addition, the 2D and 3D interaction models showed the involvement of hydrophobic interactions with Phe A99, Phe C366, Phe C407, Leu C406, Leu C415, Leu C416, and Leu C420 amino acid residues.

## 4 Discussion

Oxidative stress has been implicated in the development and prognosis of multiple diseases. To avoid oxidative stress, exogenous natural supplements can help to support the demand for endogenous antioxidants (Suleria et al., 2015). Mycoendophytes are well known for producing a variety of valuable bioactive by-products as well as their host plant. The majority of available synthesized antioxidants have been considered carcinogenic and cause liver injury (Yuan et al., 2008). By contrast, natural antioxidants produced by plants and their fungal endophytes, have not been detrimental (Caicedo et al., 2019). Polyphenols have hydroxyl groups and play a vital role in antioxidation, scavenging free radicals, and other pharmacological activities (Gangwar et al., 2014). Therefore, the discovery of polyphenol-producing endophytic fungi could help meet the needs of the pharmaceutical and food industry (Tang et al., 2021).

In the current study, ten dominant fungal endophytes were derived from black seeds belonging to ten different plant taxa. These isolates were *A. amstelodami* from *O. basilicum*, *A. niger* from *A. ampeloprasum* and *N. sativa*, *A. versicolor* from *P. nigrum*, *C. madrasense* from *P. oleracea*, *P. chrysogenum* from *A. cepa*, *P. maritimum* and *P. somniferum*, *P. citrinum* from *A. retroflexus*, and *R. oryzae* from *D. viscose*. Our results were in agreement with Metwaly et al. (2019), who isolated *Aspergillus tubingensis* which belongs to section *Nigri*, from *N. sativa* seeds. Similarly, Mandlaa et al. (2019) isolated endophytic *C. globosum* from *P. oleracea* roots. Metwaly et al. (2019) derived *A. parasiticus, Eurotium pseudoglaucus*, and *Alternaria pluriseptata* from *N. sativa* seeds, while *Penicillium*, *Alternaria*, and *Cladosporium* endophytic genera were identified by Gopane et al. (2021) from *N. sativa* seeds. Moreover, endophytic *Purpureocillium lilacinum* was obtained from seeds of *P. somniferum* (Pandey et al., 2016), and endophytic *Alternaria* spp. was recovered from *Amaranthus hybridus* seeds (Blodjett et al., 2000). However, Sreeja et al. (2019) reported that the endocarp of black pepper seed is free from fungal endophytes.

The most potent anti-free radical isolates were identified as *A. niger* TU 62, *C. madrasense* AUMC14830, and *R. oryzae* AUMC14823 by utilizing the ITS region sequencing. The ITS locus is a mostly credible region for identifying strains at species level. Molecular identification of *Aspergillus* strains to species level by ITS region sequencing was recently introduced as an effective alternative method for their accurate identification (Mazrou et al., 2020).

The present results proved that the obtained *n*-butanol extracts of both black seeds and their derived-mycoendophytes had higher content of total phenolics, flavonoids, and antioxidant capacity than acetone and methanol extracts, except *O. basilicum*, *P. maritimum*, and *A. retroflexus*, along with their fungal endophytes. The function of an antioxidant is to intercept and react with free radicals at a rate faster than the substrate (Garcia-Estan, 2019). The reduction activity of phenolic and flavonoid compounds depends on the number of free hydroxyl groups in the molecular structure, which would be strengthened by steric hindrance (Saxena et al., 2012; Platzer et al., 2022). The alcoholic extract of *P. oleracea* seeds exhibited good antiradical efficiency, and can ameliorate the H_2_O_2_ that induces death in hepatic cells (Mousavi et al., 2015; Al-Sheddi et al., 2016). Furthermore, Guo et al. (2016) reported that the oil from *P. oleracea* seed oil can be utilized as an alternate to synthetic antioxidants in food preservation and also as an ingredient in cosmetics. At the same time, Ercisli et al. (2008) and Alam et al. (2014) reported a potent antiradical activity of purslane leaves’ methanolic extract.

Our study found positive correlations as well as significant correlations between total phenolics, flavonoids, and the antioxidant activities of different organic solvent extracts from plant seeds and their derived mycoendophytes. In the same context, extraction of endophytic *P. oxalicum*, *Simplilium* sp., and *Colletotrichum* sp. culture filtrates derived from *Ligusticum chuanxiong* using different solvents (ethyl acetate, n-butanol, petroleum ether, and chloroform) showed a variation in total phenolic contents. *P. oxalicum* and *Simplicillium* sp. ethyl acetate extract was the most effective solvent for polyphenol extraction (94.58 and 58.64 mg GAE/g, respectively), while *Colletotrichum* sp. *n*-butanol was the more effective solvent for the extraction of polyphenols with 58.96 GAE/g polyphenols. In addition, antioxidant activities seemed to be correlated with total phenolic content (Tang et al., 2021). On the other hand, the methanolic extract of *Cochliobolus* sp. AL24 endophytic fungus from *Aerva lanata* exerts potent antioxidant effect, while a moderate activity was found in the dichloromethane extract followed by the butanol extract. The antioxidant activities were proportionally correlated with their total phenolic and flavonoid contents (Shoba and Sathiavelu, 2018). Conversely, Lim et al. (2021) found incompatible correlation between antioxidant potency and phenolic content.

Polar and medium polarity solvents are more favorable to the extraction of low and high molecular weight polyphenols in endophytic fungus fermentation broth than nonpolar solvents (Tang et al., 2021). Successive *n-*butanol fraction, viz., n-hexane, chloroform and ethyl acetate fractions obtained from the *Trichoderma viride* culture filtrate proved the most effective in retarding the growth of pathogenic *Alternaria alternata* afforded by *n-*butanol fraction. When these organic solvents were successively employed, organic compounds from fungal metabolites with different polarities were dissolved in different solvents and thus became separated. The disparity in bioactivity of extracts in different solvents may be ascribed to the different chemical nature of the solvents. It is the dissolution of different types of chemicals in different solvents that ensured the variable activity of metabolites of the same source in different solvents (Shafique et al., 2019). The antioxidant activity for the n-butanol and ethyl acetate extracts of *Senna italic* against the ABTS radical was 85.7 and 82.9%, respectively, compared to ascorbic acid with 89.2% inhibition. The structure elucidation of the isolated compounds from the n-butanol extract showed the presence of quercetin and rutin (Khalaf et al., 2019). Alkaloids and flavonoids present in high concentration in the *n*-butanol extract of *Apium graveolens* seeds act as an antioxidant in streptozotocin-induced diabetic mature male rats (Middleton et al., 2000; Al-Saaidi et al., 2012).

The IC_50_ values were determined for *n-*butanol extracts and showed the highest antioxidant potency of both seeds and their companion endophytic fungi using DPPH, H_2_O_2_ and nitric oxide assays. Our results showed significant positive correlations between different antioxidant activity assays of *n-*butanol extracts from plant seeds and associated mycoendophytic strains. Endophytic *A. tubingensis* from *N. sativa* seeds revealed antioxidant activity with IC_50_ value of 8 μg/ml determined by the DCFH-DA (20,70-dichlorofluorescein diacetate) method in myelomonocytic HL-60 cells (Metwaly et al., 2019). The ethanol extract of *P. maritimum* fruits showed an IC_50_ value of 6.9 μ g/mL ABTS scavenging capacity (Leporini et al., 2018). The IC_50_ values of DPPH antioxidant activity of the essential oils in Indigenous and Kerala *Piper nigrum* seed cultivars were found to be 44.16 and 22.88 mg/ml, respectively (Abukawsar et al., 2018). Moreover, a promising IC_50_ value as compared to positive standard compounds was achieved by endophytic *Aspergillus* sp. extracts isolated from *O. basilicum* var. *thyrsiflora* (Atiphasaworn et al., 2017). DPPH free radical scavenging assay is a basic and most widely used assay and considered the most accurate screening method used to evaluate the antioxidant activity of samples (Ramesha et al., 2015). However, compared to the potency of the standard antioxidants, the extracts of the endophytic fungi showed lower antioxidant activity. Moreover, the antioxidants used as standards are purified molecules, while the endophytic fungi extracts represent a group of mixtures containing different concentrations of substances (Tang et al., 2021).

According to the HPLC analysis, the different extracts of host plants and their endophytic fungi contained 17 polyphenolic compounds. The *N. sativa* extract revealed the presence of pyrocatechol as a major compound (75.79%), reported for the first time in *N. sativa*, while its endophytic fungus *A. niger* showed the presence of catechol in a small amount (3.78%), and cinnamic acid was the predominant compound in the *A. niger* extract*.* A HPLC–UV–MS study by Toma et al. (2015) reported the presence of ferulic acid, quercetin, and kaempferol in the *N. sativa* extract which supports our results. The chemical analysis revealed the presence of ellagic acid 28.47%, kaempferol (25.67%), and quercetin (23.55%) as main components beside rutin and catechin for the first time from the extract of *P. oleracea*. Chlorogenic acid (38.34%) and methyl gallate (34.41%) were found in *C. madrasense* endophyte and not detected in the host plant, indicating that these may be produced through biotransformation of host metabolites. Kaempferol and apigenin were previously reported from leaf and stem, while luteolin, myricetin and quercetin were isolated from the whole *P. oleracea* plant (Zhou et al., 2015). Salucci et al. (2002) reported that dietary flavonoids like epicatechin, galate, gallic acid, and quercetin-3-glucoside exhibit strong antioxidant activity. The dietary flavonol, quercetin, is a potent antioxidant because it has all the right structural features for free radical scavenging activity (Formica and Regelson, 1995). Quercetin, kaempferol, morin, myricetin and rutin, by acting as antioxidants, exhibited beneficial effects such as anti-inflammatory, antiallergic, antiviral, as well as anticancer activity (Saxena et al., 2012).

Our results revealed that hesperetin and syringic acid were the major detected compounds (27.69 and 21.43%, respectively) in the *D. viscosa n-*butanol extract, while the endophytic fungi *R. oryzae* extract showed the presence of caffeic acid (33.22%) as a major compound, which was not detected in the host plant. Previous studies on *D. viscosa* reported the presence of alkaloids, flavonoids, phenols, steroids, glycosidic cyanide, saponins and tannins in which flavonoids and phenols were the major components in accordance with our results (Alzandi et al., 2021). Ghisalberti (1998) identified 23 flavones from seeds, bark, flowers and leaves of *D. viscosa*, characterized at c-3 and in almost 50% of cases, methoxylation at c-6. It is noteworthy that plant-endophyte metabolisms can interact by inducing each other’s metabolisms or metabolizing secondary compounds from each other *via* different biosynthetic and biotransformation pathways (Ludwig-Muller, 2015). These reports could explain the variance between the detected compounds in the host plant and their derived mycoendophytes in our study.

The binding interaction and conformation of each phytocompound with each target protein was ranked based on lowest energy and lowest RMSD, respectively, according to previous studies that indicated that the lower the binding energy score found, the better the protein–ligand binding stability (Imana et al., 2020). The investigation of molecular simulation results of detected phytomolecules against myeloperoxidase enzymatic protein (1DNU chain A & C) revealed that rutin possess high affinity for the enzyme (−15.3184 kcal/mol) to act as inhibitor. Rutin (7 ± 1 mg/kg dw) was determined in *A. retroflexus* seeds (Kalinova and Dadakova, 2009). Rutin is a flavonol that has exhibited a variety of pharmacological efficacies including antioxidant, cytoprotective, anticarcinogenic, neuroprotective, and cardioprotective activities (Ganeshpurkar and Saluja, 2017; Enogieru et al., 2018). Flavonoids have the hydroxyl groups and functional substitutions that provide their bioactivity and have an important role in non-covalent interactions such as electrostatic interactions, hydrogen bonds, van der Waals interactions, and hydrophobic interactions with their targets in drug-enzyme interactions and are critical in drug design (Comakli et al., 2020). Phenolic structures often have the potential to strongly interact with proteins, due to their hydrophobic benzenoid rings and the hydrogen-bonding potential of the phenolic hydroxyl groups. This gives phenolics the ability to act as antioxidants also by virtue of their capacity to inhibit some enzymes involved in radical generation (Pereira et al., 2009).

## 5 Conclusion

In this study, ten mycoendophytes associated with black seed plants, and their *n*-butanol, acetone, and methanol crude extracts, demonstrated different levels of total phenolics and flavonoids, in addition to antiradical bioactivity. *A. niger* TU 62, *C. madrasense* AUMC14830, and *R. oryzae* AUMC14823 derived from *N. sativa*, *P. oleracea*, and *D. viscosa* seeds, respectively, as well as the *n*-butanol extracts, showed the highest antiradical potency. Moreover, assayed *n*-butanol extracts demonstrated significant IC_50_ values as well as significant positive correlations between different antioxidant activity assays. In HPLC analysis of the most antiradical efficient *n*-butanol extracts, multiple phenolic and flavonoid compounds were detected in different degrees of existence and concentration. Furthermore, molecular docking of the investigated molecules demonstrated that rutin held high affinity against myeloperoxidase and could act as an inhibitor. Concerning the obtained results, the mycoendophytes associated with black seed plants might be deemed valuable sources for the high-scale industrial production of antioxidants, given their virtue therapeutic prospects and lack of any adverse effects.

## Data Availability

The raw data supporting the conclusion of this article will be made available by the authors, without undue reservation.
